# Structural and Functional Evaluation of *C. elegans* Filamins FLN-1 and FLN-2

**DOI:** 10.1371/journal.pone.0022428

**Published:** 2011-07-25

**Authors:** Christina R. DeMaso, Ismar Kovacevic, Alper Uzun, Erin J. Cram

**Affiliations:** 1 Department of Biology, Center for Interdisciplinary Research on Complex Systems, Northeastern University, Boston, Massachusetts, United States of America; 2 Department of Pediatrics, Women and Infants Hospital of Rhode Island, Brown Alpert Medical School, Center for Computational Molecular Biology, Brown University, Providence, Rhode Island, United States of America; Brown University, United States of America

## Abstract

Filamins are long, flexible, multi-domain proteins composed of an N-terminal actin-binding domain (ABD) followed by multiple immunoglobulin-like repeats (IgFLN). They function to organize and maintain the actin cytoskeleton, to provide scaffolds for signaling components, and to act as mechanical force sensors. In this study, we used transcript sequencing and homology modeling to characterize the gene and protein structures of the *C. elegans* filamin orthologs *fln-1* and *fln-2*. Our results reveal that *C. elegans* FLN-1 is well conserved at the sequence level to vertebrate filamins, particularly in the ABD and several key IgFLN repeats. Both FLN-1 and the more divergent FLN-2 colocalize with actin *in vivo*. FLN-2 is poorly conserved, with at least 23 IgFLN repeats interrupted by large regions that appear to be nematode-specific. Our results indicate that many of the key features of vertebrate filamins are preserved in *C. elegans* FLN-1 and FLN-2, and suggest the nematode may be a very useful model system for further study of filamin function.

## Introduction

Filamins are long, flexible, multi-domain proteins composed of an N-terminal actin-binding domain (ABD) followed by multiple immunoglobulin-like repeats (IgFLN). The best-characterized filamins are *Dictyostelium discoideum* filamin (ddFLN) and human filamins (hsFLNA/B/C). *Dictyostelium* filamin has an ABD followed by six IgFLN repeats, whereas the human orthologs have 24 IgFLN repeats arranged into two rod domains separated by a flexible hinge. FLNA, FLNB, and FLNC are more than 70% identical at the amino acid sequence level and have overlapping expression patterns. Although FLNA and FLNB are ubiquitously expressed, FLNC is found primarily in cardiac and striated muscle [1]. Filamins are involved in diverse cellular processes including anchoring, organizing and maintaining the actin cytoskeleton, providing a scaffold for signaling components, and acting as molecular sensors for mechanical forces [1]. Due to the pleiotropic functions of filamins in humans, mutations cause a wide variety of developmental defects in the skeleton, brain, heart, and smooth muscle [2].

Although no complete structure of a filamin molecule is available, biochemical and structural studies have provided important insights into the function of filamins [3], [4], [5]. The best-studied role of filamin is in the organization of actin filaments into branched three-dimensional networks [1]. Filamin binds F-actin using the N-terminal ABD, although some IgFLN repeats and hinge regions may also contribute to actin binding [6]. The filamin ABD consists of two calponin homology (CH) domains that are well conserved among filamins and other actin binding proteins, such as alpha-actinin, spectrin, and fimbrin [7]. In filamin, the primary actin-binding site is hydrophobic and is located in the first CH domain (CH1) [8], [9], [10]. The second CH domain (CH2) has a lower affinity for actin, but is required for a fully functional ABD [10], [11]. Although CH2 is less conserved across filamins than CH1, disease-related mutations suggest that CH2 may regulate the actin-binding activity of CH1 [12]. For example, gain-of-function mutations in the CH2 domain of FLNA lead to developmental disorders of the skeleton by increasing filamin affinity for F-actin, which perturbs actin cytoskeleton dynamics [13].

Individual IgFLN repeats are ∼96 amino acids in length and are comprised of seven β-strands (A–G) arranged into two β-sheets, which together form a β-sandwich. Filamins are predicted to interact with more than fifty different proteins, many of which interact with the CD strands of the IgFLN domains [14]. The majority of these interactions involve IgFLN domains in the second rod domain (IgFLN16–24). For example, filamin binds transmembrane proteins such as integrins [15], transmembrane receptors [16], and many signaling proteins, including the Rho-family of GTPases [17], [18]. The cytoplasmic tail of β7 integrin binds to the CD face of FLNA IgFLN21 [5], which links the actin network physically with the extracellular matrix (ECM). FLNA IgFLN24 binds RhoA, Rac1 and Cdc42, all of which regulate actin dynamics. In addition, the final repeat also mediates dimerization of filamins [6], [19], [20]. FLNB has also been shown to serve as a scaffold for signaling pathway components, for example, the Rac1, MEKK1, MKK4, and JNK cascade in interferon-induced apoptosis [1], [21], [22].

We are using the nematode *C. elegans* as a model system to study the conserved functions of filamin *in vivo*. *C. elegans* provides many advantages for the study of cytoskeletal regulation, including a translucent body, good visualization tools, and freely available genomic resources [23]. The *C. elegans* genome encodes two filamin homologs, *fln-1* and *fln-2*. In addition to filamins, many other cytoskeletal regulators are well conserved, including integrin [24], talin [25], vinculin [26], and the Rac GTPases [27]. We have recently shown that FLN-1 is required for maintenance of actin and for proper function of the spermatheca, a somatic tissue of the nematode reproductive system that undergoes dramatic shape changes during the ovulation and fertilization of oocytes [28]. The second *C. elegans* filamin ortholog, FLN-2, has not been studied, but large-scale RNAi studies suggest it may play a role in molting [29], [30]. In this study, we characterize the gene structures and expression patterns of *fln-1* and *fln-2,* use homology modeling to determine the structure of the conserved IgFLN domains in these molecules, predict the conservation of function between *C. elegans* and other filamins, and demonstrate that the ABD of FLN-1 and FLN-2 can co-localize with actin *in vivo*.

## Results

### The *C. elegans* genome encodes two filamin genes, *fln-1* and *fln-2*

**Figure 1:**
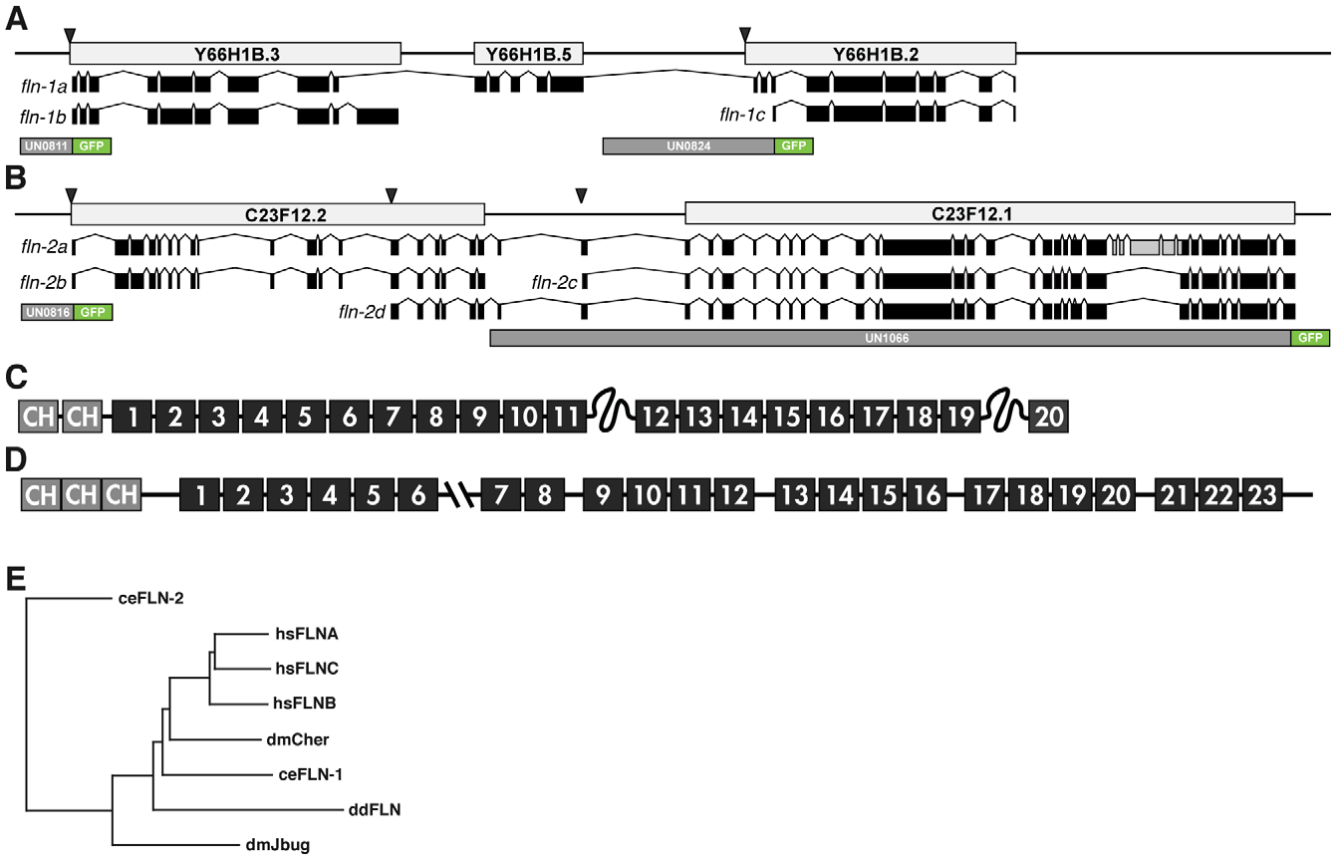
Gene structure of FLN-1 and FLN-2 filamin. A schematic representation of the *fln-1* (A) and *fln-2* (B) loci. Light grey boxes indicate the genomic region of the predicted ORFs. Exons determined by cDNA sequencing are shown in black, and introns are shown as thin lines. SL1 trans-splice acceptor sites are indicated with arrowheads. Green exons in *fln-2a* indicate exons that are predominantly spliced out. Schematic representation of the domain structure of FLN-1A (C) and FLN-2A (D) as determined by sequence alignment with human FLNA. The first and second rod regions of FLN-1 are colored blue and orange, respectively. (E) *C. elegans* FLN-1 clusters with human filamins, the *Drosophila* filamin cheerio, and *Dictyostelium* filamin, while *C. elegans* FLN-2 most closely resembles *Drosophila* filamin jitterbug.

In order to identify filamin-like genes in *C. elegans*, we performed a BLAST search of the *C. elegans* genome (WormBase release WS190) using human FLNA. At the time, this search identified a cluster of predicted open reading frames (ORF) on chromosome IV (Y66H1B.2, Y66H1B.5, and Y66H1B.3; Figure 1A) and another on chromosome X (C23F12.1 and C23F12.2; Figure 1B). These ORFs lack one or more of the characteristic features of filamin, the ABD or IgFLN repeats, but taken together appeared to encode proteins with a domain structure similar to other filamins. To determine which transcripts are produced from these loci, we performed RT-PCR on total RNA extracted from mixed-stage wild-type (N2) nematodes, and sequenced the resulting cDNAs (Figure S1). Sequencing of cDNAs derived from the *fln-1* and *fln-2* loci allowed us to identify three *fln-1* isoforms and four *fln-2* isoforms (Figures 1 and S1, Table 1). Sequences spanning Y66H1B.2, Y66H1B.3 and Y66H1B.5 were readily detected, suggesting these three ORFs are transcribed together and represent a single filamin locus. Similarly, C23F12.2 and C23F12.1 were found to comprise a second filamin locus. The names *fln-1* and *fln-2* have been assigned to the Y66H1B and C23F12 filamin loci, respectively, and the amended gene models are available in WormBase.

**Table 1:** Splice variants of *fln-1.*

Isoform	Former Sequence IDs*	New Sequence ID	Size (aa)	Domains
FLN-1A	Y66H1B.2, Y66H1B.3, Y66H1B.5	Y66H1B.2a	2257	ABD, 20 Ig-FLN
FLN-1B	Y66H1B.3	Y66H1B.2b	1084	ABD, 8 Ig-FLN
FLN-1C	Y66H1B.2	Y66H1B.2c	836	9 Ig-FLN

*Former sequence IDs are based on WormBase WS205, while the new sequence IDs are based on WormBase WS210.

To identify the 5′ ends of the transcripts we took advantage of SL1 trans-splicing in nematodes. The majority of transcripts in *C. elegans* are trans-spliced to a 22-nucleotide splice leader sequence, SL1 [31]. The SL1 sequence is commonly used in *C. elegans* to identify the 5′ ends of transcripts [32]. We performed RT-PCR using SL1 and gene-specific primers, and sequenced the resulting amplicons. Y66H1B.3, Y66H1B.2, C23F12.1, and two independent C23F12.2 transcripts, are trans-spliced to SL1, which suggests that each of these ORFs can be transcribed independently (Figures 1 and S1). The predicted ORF Y66H1B.5 is not trans-spliced to SL1, and is probably only found as part of full-length *fln-1*.

**Figure 2:**
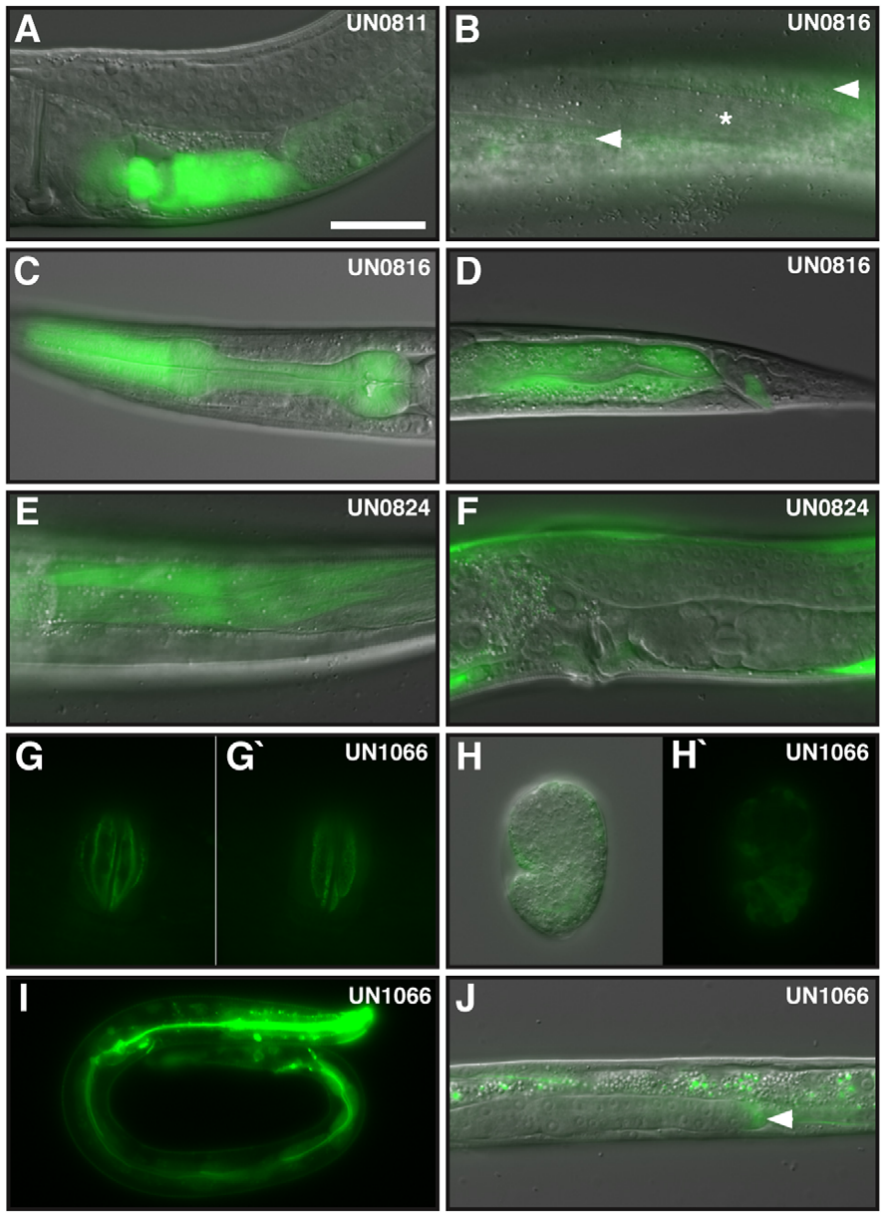
Expression of *fln-1* and *fln-2*. Merged DIC and GFP images of a young adult hermaphrodite carrying (A) a transcriptional *fln-1a::gfp* fusion expressed in the uterus, spermatheca and sheath cells. Images (B-D) depict a transcriptional *fln-2a::gfp* fusion expressed in the (B) hypodermis (arrowhead) but not muscle cells (asterisk), (C) pharynx, (D) intestine and anal depressor muscle. Images (E, F) depict a transcriptional *fln-1c::gfp* fusion expressed in (E) body wall muscle and (F) hypodermis and vulval muscle. Images (G–J) depict a translational *fln-2c::gfp* fusion. Images (G) and (G’) are two different focal planes of *fln-2c::gfp* expressed in the vulva. Images (H) and (H’) depict *fln-2c::gfp* in hypodermal cells of a comma stage embryo. Image (I) depicts *fln-2c::gfp* in hypodermal cells, intestine and pharynx of an L1 larva. Image (J) depicts *fln-2c::gfp* in the DTC (arrowhead) of an L2 larva. Scale bar is 25 µm.

*fln-1a*, *b*, and *c* are the three major *fln-1* transcripts, with *fln-1a* representing a full-length filamin that includes an actin-binding domain and 20 Ig-like filamin repeats (Figures 1A and S1A). We are able to readily detect the full-length (6.7 kb) transcript from wild-type nematodes using RT-PCR. The structure of the FLN-1A isoform is similar to that that predicted by Heikkinen, et al. [33]. The *fln-1b* and *fln-1c* transcripts are the result of alternative splicing and an alternate transcriptional start site, respectively. *fln-1b* includes an exon that is not a part of the full-length *fln-1a* (Figure 1A and S1A). We also detected rare transcripts spanning Y66H1B.3 and Y66H1B.2, but did not include Y66H1B.5, some of which contained stop codons at the beginning of Y66H1B.2 due to alternative splicing (Figure S1). Analysis of GFP reporter constructs (Figure 1A) suggests *fln-1a*, and likely *fln-1b*, are predominantly expressed in the somatic gonad, including the spermatheca, sheath, and uterus (Figure 2A). We have shown previously that a full length, functional FLN-1A::GFP fusion protein is expressed and co-localizes with actin filaments in these tissues [28]. In contrast, the *fln-1c* construct is expressed in the body wall muscle, vulval muscle and hypodermis (Figure 1E,F).

*fln-2a* represents the longest predicted transcript from the *fln-2* locus based on cDNA sequencing (Figure 1B and S1B); however, we were unable to detect the full-length *fln-2a* transcript by PCR, likely because of the transcript size (>11 kb). *fln-2b* is the result of alternative splicing in the final exon of C23F12.2, which introduces a stop codon, while *fln-2c* and *d* are the result of alternative transcriptional starts (Figure 1B). In addition to the *fln-2* SL1 sites we identified, the modENCODE consortium has identified numerous SL1 sites within C23F12.2 [34]. This suggests that *fln-2* may have many transcriptional start sites. These additional SL1 sites would produce transcripts similar to *fln-2c* and *d*. Based on RNA-seq expression data from modENCODE it seems likely that *fln-2a* and *b* are only expressed during embryogenesis, while *fln-2c* and *d* are expressed throughout the life stages. In addition, five exons near the 3′ end of *fln-2*, colored light gray in Figure 1B, are usually excluded, suggesting that most transcripts are lacking these five exons. Analysis of a series of GFP reporter constructs (Figure 1B) suggests the various isoforms of *fln-2* are expressed broadly, with strong expression in the hypodermis, pharynx, intestine, anal depressor muscle and distal tip cell (Figure 2C–D, G–J). The modENCODE data suggests that the FLN-2C isoform might be the most strongly expressed version of FLN-2. To determine the expression pattern and subcellular localization of FLN-2C, we constructed a translational GFP fusion. FLN-2C::GFP localizes in small puncta at the cell membranes of vulval (Figure 2G) and hypodermal cells (Figure 2H,I) and to the intestinal lumen (Figure 2I). Lacking a mutant allele of *fln-2* with an overt phenotype, we are unable to determine the functional significance of these expression patterns.

To visualize evolutionary relationships between the *C. elegans* filamins and well-studied filamins from humans, *Drosophila* and *Dictyostelium*, we constructed a phylogenetic tree using the neighbor joining method [35]. Human filamins, *Drosophila* filamin *cheerio*, *C. elegans* filamin FLN-1, and *Dictyostelium* filamin cluster together (Figure 1E). There is insufficient homology to indicate to which human filamin FLN-1 is most similar. In contrast, FLN-2 is a less well-conserved filamin, distant even from the divergent *Drosophila* ortholog *jitterbug*.

### Domain identification of *C. elegans* filamins

In order to identify the actin-binding and IgFLN domains in FLN-1 and FLN-2, we used the sequence feature scan in SWISS-MODEL Workspace [36], the Simple Modular Architecture Research Tool (SMART) [37], [38], and searched the protein sequence for conserved SPF motifs near the ends of IgFLN repeats [39]. (See figure S2 for an alignment of FLN-1 IgFLN repeats). The full-length form, *fln-1a*, is predicted to encode a 2255 amino acid protein composed of an N-terminal ABD, followed by 20 IgFLN repeats (Figure 1C, Table 1). The *fln-1b* transcript encodes the ABD and 8 IgFLN repeats, two of which are alternatively spliced and not found in *fln-1a*. Finally, the *fln-1c* transcript is predicted to encode only the final 9 IgFLN repeats (Table 1). The full-length isoform, *fln-2a*, encodes a 3747 amino acid protein composed of an N-terminal ABD followed by at least 23 IgFLN domains (Figure 1D, Table 2). The FLN-2B protein consists of an ABD followed by a 300 amino acid low-homology region and two IgFLN repeats. FLN-2C and FLN-2D proteins contain IgFLN repeats, but do not have an ABD (Figure 1, Table 2).

**Table 2:** Splice variants of *fln-2.*

Isoform	Former Sequence IDs*	New Sequence ID	Size (aa)	Domains
FLN-2A	C23F12.1, C23F12.2	C23F12.1a	3747	ABD, ∼23 Ig-FLN
FLN-2B	C23F12.2	C23F12.1b	861	ABD, 2 Ig-FLN
FLN-2C	C23F12.1	C23F12.1c	2851	∼20 Ig-FLN
FLN-2D	C23F12.1, C23F12.2 (partial)	C23F12.1d	3128	∼23 Ig-FLN

*Former sequence IDs are based on WormBase WS205, while the new sequence IDs are based on WormBase WS210.

### Homology modeling of IgFLN repeats in FLN-1 and FLN-2

**Figure 3:**
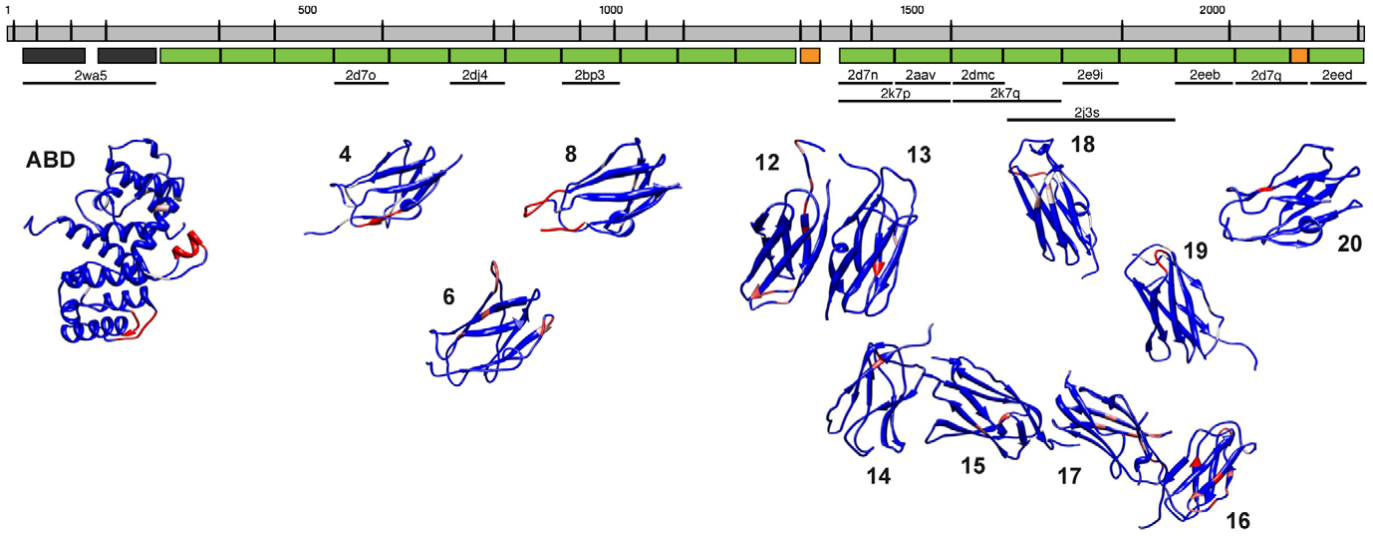
Homology modeling of FLN-1. The spliced *fln-1a* sequence is illustrated by the gray rectangles (introns are not shown). IgFLN domains determined by sequence alignment with human FLNA are shown as green rectangles, hinge regions are shown as orange rectangles, and CH domains are shown as dark gray rectangles. Structural templates are indicated by thick black lines, and labeled with the PDB ID. The modeled ABD and the IgFLN structures are shown below. The structures are colored to reflect average B-factor, with red regions being more flexible than blue regions.

**Figure 4:**
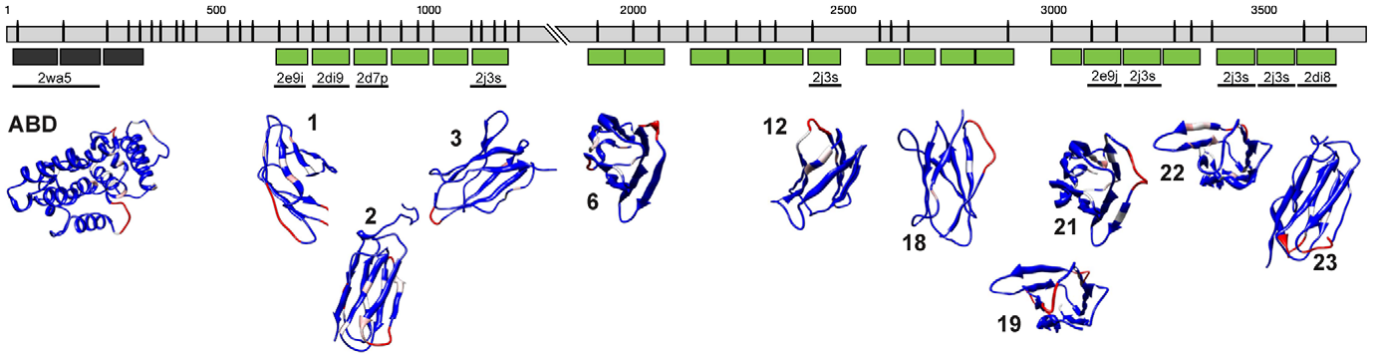
Homology modeling of FLN-2. The spliced *fln-2a* sequence is illustrated by the gray rectangles (introns are not shown). IgFLN domains determined by sequence alignment with human FLNA are shown as green rectangles, and the CH domains are shown as dark gray rectangles. Structural templates are indicated by thick black lines, and labeled with the PDB ID. The modeled ABD and the IgFLN structures are shown below. The structures are colored to reflect average B-factor, with red regions being more flexible than blue regions.

We performed homology modeling of the *C. elegans* filamins using the SWISS-MODEL Workspace [36], [40]. The human filamin actin-binding domain and many of the IgFLN repeats have structures available in the Protein Data Bank (PDB). We identified templates, and used ClustalW [41], as well as manual adjustments, to refine template alignment to the target sequence before modeling. We used stringent criteria for homology modeling, retaining only those models with a sequence identity greater than 30% and a QMEAN score greater than 0.500 [42]. For FLN-1, 13 of the 21 domains met these criteria (Table 3, Figure 3), while 11 of the 24 FLN-2 domains possessed sufficient homology for modeling (Table 4, Figure 4). The QMEAN analysis indicates that experimentally determined IgFLN structures for FLN-1 and FLN-2 are statistically likely to be similar to these predicted models [42].

**Table 3:** Modeling statistics for FLN-1.

FLN-1 Domain	Region (aa)	AA	PDB Template	Identity	QMEAN Model	QMEAN Template	Z-score Model	Z-score Template
CH1-CH2	15–252	236	2WA5A (hsFLNB ABD)	54.0%	0.683	0.756	−0.071	0.060
IgFLN04	545–635	91	2D7OA (hsFLNC Ig17)	43.5%	0.707	0.739	−0.280	0.110
IgFLN06	736–828	93	2DJ4A (hsFLNB Ig13)	38.9%	0.797	0.862	0.480	1.260
IgFLN08	926–1019	94	2BP3A (hsFLNA Ig17 GPIBa)	45.2%	0.636	0.724	−0.900	−0.140
IgFLN12	1396–1474	79	2D7NA (hsFLNC Ig16)	34.6%	0.882	0.863	1.090	1.040
IgFLN13	1476–1568	93	2AAVA (hsFLNA Ig17)	46.7%	0.846	0.818	0.890	0.720
IgFLN14	1569–1654	86	2DMCA (hsFLNB Ig18)	37.2%	0.902	0.677	1.360	−0.480
IgFLN15	1655–1752	98	2J3SA (hsFLNA Ig19)	49.0%	0.804	0.651	0.600	−0.900
IgFLN16	1754–1874	94	2E9IA (hsFLNB Ig20EX)	31.9%	0.814	0.834	0.610	0.980
IgFLN17	1848–1941	94	2J3SA (hsFLNA Ig21)	56.5%	0.706	0.651	−0.290	−0.900
IgFLN18	1942–2039	97	2EEBA (hsFLNB Ig22)	31.6%	0.770	0.924	0.260	1.830
IgFLN19	2040–2131	92	2D7QA (hsFLNC Ig23)	55.3%	0.787	0.840	0.380	1.100
IgFLN20	2162–2254	89	2EEDA (hsFLNB Ig24)	43.0%	0.706	0.719	−0.270	−0.140
			average:	43.6%	0.772	0.774	0.297	0.349

Only repeats with a sequence identity greater than 30% to an available structure in the PDB are shown. AA is number of amino acids. Average sequence identity, QMEAN, and Z-scores are shown at the bottom.

**Table 4:** Modeling statistics for FLN-2.

FLN-2 Domain	Region (aa)	AA	PDB Template	Identity	QMEAN Model	QMEAN Template	Z-score Model	Z-score Template
CH1-CH2	16–226	211	2WA5A (hsFLNB ABD)	43.3%	0.787	0.756	0.360	0.060
IgFLN01	641–717	77	2E9IA (hsFLNB Ig20EX)	31.6%	0.868	0.834	0.990	0.980
IgFLN02	722–821	100	2DI9A (hsFLNB Ig9)	29.1%	0.869	0.668	0.990	−0.630
IgFLN03	823–915	93	2D7PA (hsFLNC Ig22)	32.3%	0.681	0.923	−0.480	1.940
IgFLN06	1108–1196	89	2J3SB (hsFLNA Ig19)	30.4%	0.705	0.651	−0.290	−0.900
IgFLN12	2418–2497	80	2J3SA (hsFLNA Ig21)	30.0%	0.755	0.651	0.160	−0.900
IgFLN18	3073–3163	91	2E9JA (hsFLNB Ig14)	32.3%	0.787	0.749	0.400	0.240
IgFLN19	3164–3255	92	2J3SA (hsFLNA Ig19)	31.2%	0.731	0.651	−0.080	−0.900
IgFLN21	3390–3484	95	2J3SB (hsFLNA Ig19)	33.7%	0.640	0.651	−0.860	−0.900
IgFLN22	3486–3578	93	2J3SA (hsFLNA Ig19)	38.0%	0.672	0.651	−0.580	−0.900
IgFLN23	3580–3675	96	2DI8A (hsFLNA Ig19)	39.6%	0.783	0.769	0.390	0.400
			average:	33.8%	0.753	0.723	0.091	−0.137

Only repeats with a sequence identity greater than 30% to an available structure in the PDB are shown. AA is number of amino acids. Average sequence identity, QMEAN, and Z-scores are shown at the bottom.

In full-length FLN-1, 20 IgFLN repeats follow the N-terminal ABD (Figure 1C, Figure 3). We were able to model three IgFLN domains from the first rod domain, and all of the second rod IgFLN domains (Figure 3, Table 3). Importantly, each IgFLN repeat in the second rod domain maps to a specific IgFLN repeat in human FLNA (Figure 3, Table 3), suggesting that the domains are not just interchangeable modules, but may mediate specific and conserved interactions. In order to predict how the IgFLN domains in *C. elegans* filamins might interact with each other, we used multi-domain structures 2K7P (FLNA 16–17), 2K7Q (FLNA 18–19), and 2J3S (FLNA 19–21) as scaffolds to arrange our domain models (Figure 3). The resulting models suggest that the IgFLN repeats in the FLN-1 second rod domain would adopt an arrangement similar to those found in human FLNA.

FLN-2 is composed of an N-terminal ABD followed by at least 23 IgFLN domains, interspersed with long regions for which no structures were available (Figures 1D and 4). It is possible the low homology regions in FLN-2 encode additional IgFLN repeats. This lower overall homology resulted in fewer high-quality IgFLN domain models, and the mapping of five FLN-2 IgFLN repeats to a single PDB model 2J3S (Figure 4, Table 4). Despite these caveats, the model quality estimation parameters for the FLN-2 IgFLN repeat models are similar to those of the FLN-1 IgFLN repeats (Table 3), suggesting FLN-2 is likely to be composed primarily of filamin-like repeats. We were unable to perform multi-domain modeling for FLN-2 due to poor sequence identity with the available multi-domain templates.

### Homology modeling of the FLN-1 and FLN-2 actin binding domains

**Figure 5:**
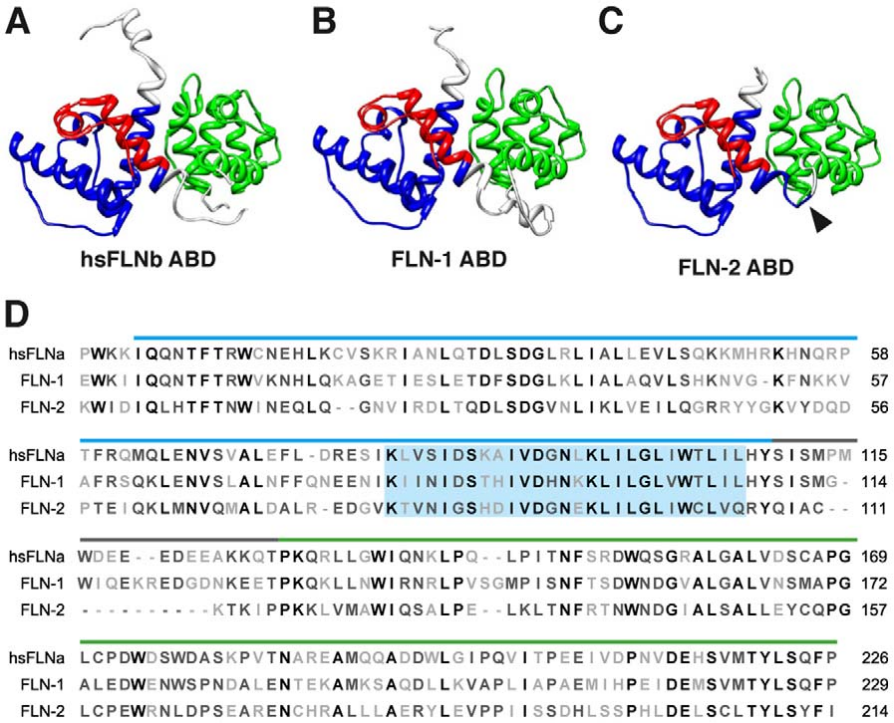
Actin-binding domain modeling of FLN-1 and FLN-2. Human FLNA ABD structure 2wa5 (A) was used to model the FLN-1 (B) and FLN-2 (C) ABDs. Arrowhead in (C) indicates missing linker region between CH1 and CH2 of FLN-2. The third CH domain of FLN-2 was not modeled. (D) Sequence alignment of human FLNA, and *C. elegans* FLN-1 and FLN-2 ABDs. CH1 and CH2 domains are indicated with blue and green, respectively. The linker region is indicated with gray. Highlighted region in (D) indicates the main actin-binding site in CH1.

We identified actin-binding domains (ABDs) near the N-termini of FLN-1 and FLN-2 (Figures 3, 4, and 5). Alignment of the ABDs of FLN-1, FLN-2, and human FLNA indicates the filamin ABDs are composed of well-conserved CH domains (Figure 5D). The CH1 and CH2 domains of FLN-1 and FLN-2 were modeled using the FLNB ABD structure (PDB ID 2WA5) [43]. The resulting FLN-1 and FLN-2 ABD models are very similar to the FLNB structure (Figure 5A–C). Both the degree of homology and statistical analysis of the models suggest the structure and function of the *C. elegans* ABDs are likely to be extremely well conserved (Tables 2 and 3). Interestingly, the FLN-2 ABD contains a third, more divergent, CH domain. No PDB structures were available with sufficient homology for modeling the FLN-2 CH3 domain. In addition, the linker region between CH1 and CH2 is absent in FLN-2, suggesting that interaction of the CH1 and the CH2 domain, and hence the actin binding activity of FLN-2 may be regulated differently (Figure 5C) [10].

### FLN-1 and FLN-2 actin binding domains colocalize with actin *in vivo*

**Figure 6:**
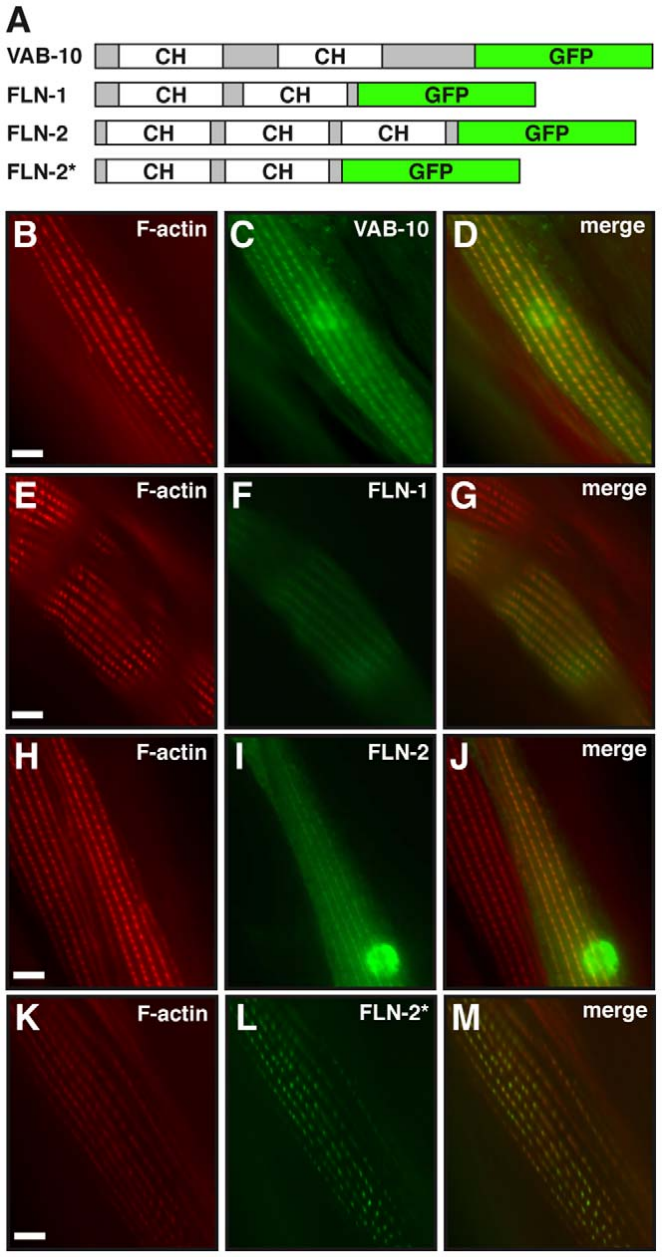
FLN-1 and FLN-2 ABD mediates co-localization with actin *in vivo*. (A) Diagrams of ABD::GFP constructs used. (B, E, H and K) Body-wall muscle cells are stained for F-actin with phalloidin. (C,F,I and L) VAB-10, FLN-1, FLN-2, and FLN-2* (CH1 and CH2) ABDs fused to GFP, and expressed in muscle cells. (D, G, J and M) Merged F-actin and GFP images show co-localization. Scale bar indicates 5 µm.

Because of the significant homology between the *C. elegans* and human filamin ABDs, and the results of our homology modeling, we predicted that FLN-1 and FLN-2 would colocalize with actin *in vivo*. We tagged the FLN-1 and FLN-2 ABD with green fluorescent protein (GFP), generated transgenic nematodes by microinjection, and used fluorescence microscopy to determine the co-localization of the GFP fusions with actin filaments in body-wall muscle cells. FLN-1 and FLN-2 ABD localization was compared to animals expressing a VAB-10/spectraplakin ABD GFP fusion protein. VAB-10, like filamin, has an ABD consisting of tandem CH domains and has been used to label actin filaments in *C. elegans* [44]. F-actin was visualized in the transgenic nematodes by staining with Texas red-conjugated phalloidin. We observed colocalizaiton of the FLN-1 and FLN-2 ABDs with F-actin (Figure 6E–J) in a manner indistinguishable from the VAB-10 fusion protein (Figure 6B–D). To determine if the third CH domain in the FLN-2 ABD is necessary for localization to actin, we generated transgenic animals in which the two most highly conserved domains (CH1 and CH2) of FLN-2 were fused to GFP. The localization pattern (Figure 6 K–M) was indistinguishable from the other ABD constructs, indicating the third CH domain is not required for localization. These data suggest that the CH1 and CH2 domains of *C. elegans* filamins can direct the localization of GFP to actin filaments, likely through direct binding to actin.

### IgFLN repeats are conserved in FLN-1

**Figure 7:**
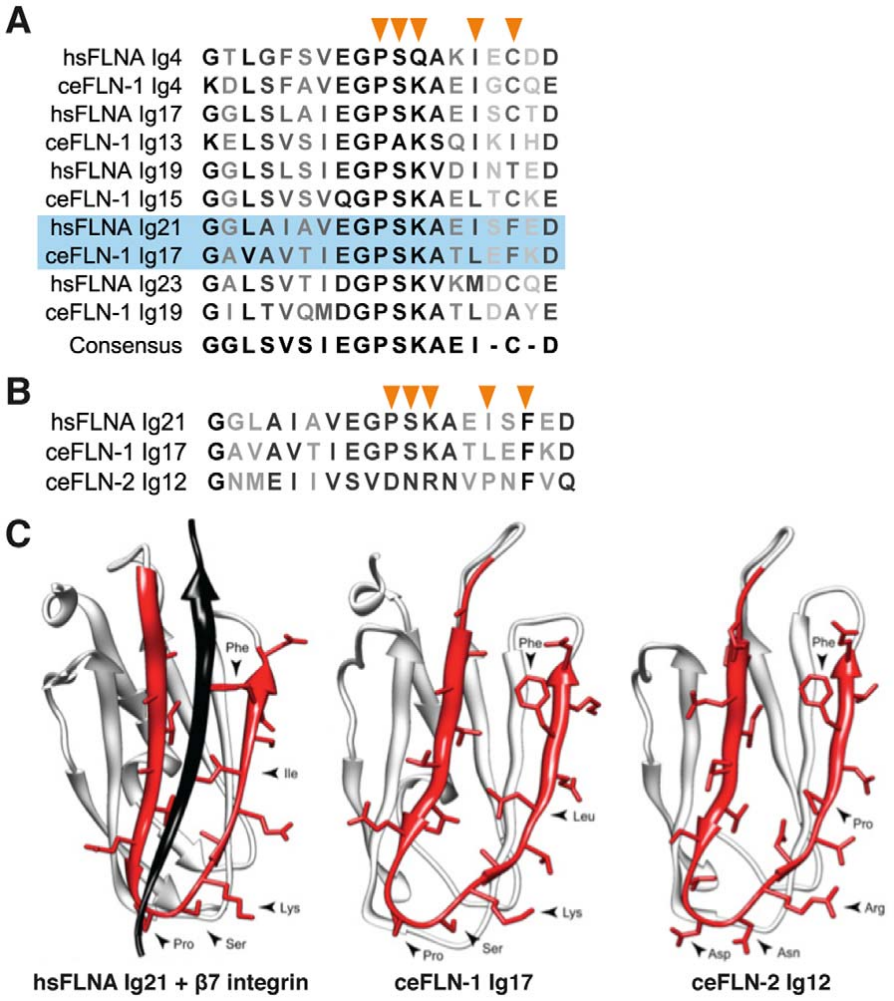
Alignment of group A CD binding interface. (A) Alignment of selected group A repeats C-D faces of human FLNA and *C. elegans* FLN-1. Residues critical for integrin-binding are indicated with orange arrowheads. FLNA IgFLN21 and FLN-1 IgFLN17 are highlighted in blue. (B) Alignment of the FLNA IgFLN21 and FLN-1 IgFLN17 C–D faces with FLN-2 IgFLN12. (C) Structure of hsFLNA IgFLN21 complexed with the β7 integrin tail, and the homology models of FLN-1 IgFLN17 and FLN-2 IgFLN12. Arrowheads indicate critical residues for integrin binding.

IgFLN domains are composed of two beta-sheets that combine to form a beta sandwich. The first sheet contains four beta strands (A, B, E, and D) and the second is composed of three strands (C, F, and G). Human IgFLN repeats have been classified into four distinct groups: A, B, C, and D [14]. Using sequence alignments and phylogenetic trees, we identified six group A repeats (4, 8, 13, 15, 17 and 19), five group B repeats (12, 14, 16, 18, and 20), and one group D repeat (6) (Figure 7A, S2, and S3). Group C and D repeats are located in the first rod of filamin and have a lower conservation compared to group A and B repeats. Importantly, not all repeats in the first rod domain are poorly conserved; namely repeats 4 and 8, which belong to group A.

Group A repeats have a conserved ligand-binding motif on the CD face and are responsible for most of the known filamin interactions [14]. Although other Group A repeats bind integrin with lower affinity, the hsFLNA IgFLN21 is the primary site of integrin binding [4], [5]. The overall structure, position in the second rod domain, and key integrin binding residues present in FLN-1 IgFLN17 very closely resemble FLNA IgFLN21, suggesting that the nematode filamin may also bind integrin in this region (Figure 7B–C). In contrast, while our modeling studies indicate FLN-2 IgFLN12 will fold similarly to FLNA IgFLN21, substitutions exist in several key residues on the CD face that would make integrin binding unlikely (Figure 7B–C) [4], [5].

**Figure 8:**
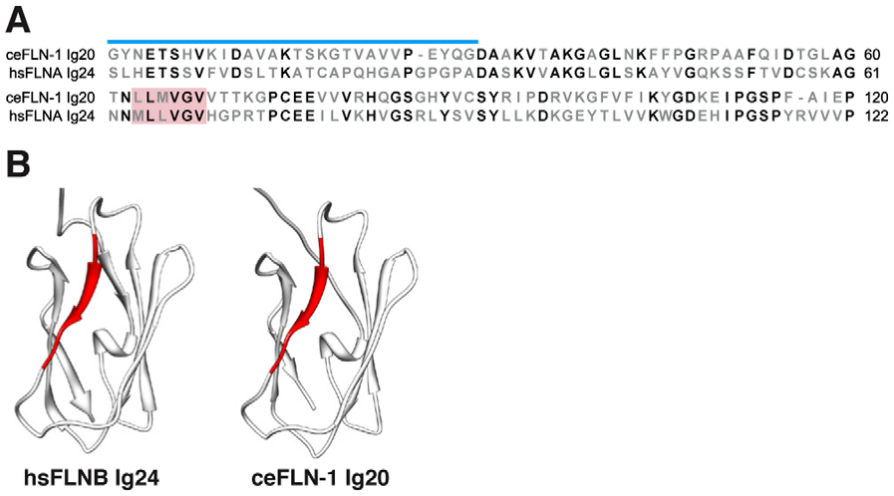
FLN-1 dimerization domain. (A) Alignment of FLNA and FLN-1 dimerization domains, with the hydrophobic dimerization interface indicated in red. Blue line denotes the hinge region between the dimerization domain and the preceding domain. (B) Structural comparison of the solution NMR structure of FLNB IgFLN24 (PDB ID 2EED) with the nearly identical homology model of FLN-1 IgFLN20. The conserved hydrophobic dimerization signature is in red. The hinge region is not shown.

*In vitro* studies of *Dictyostelium* and human filamins indicate that the C-terminal Group B repeat mediates homo- and hetero-dimerization between filamin monomers [9]. Our homology modeling results predict that the C-terminal IgFLN repeat of FLN-1 (IgFLN20) is structurally similar to that of the final human filamin repeat IgFLN24 (PDB structure 2EED), including the hydrophobic dimerization signature [45], [46] (Figure 8A–B). In contrast, the final repeat of FLN-2 does not show specific structural homology to the dimerization domain, or possess the residues predicted to mediate dimerization (Figure 4, Table 4). These modeling results suggest FLN-1 and FLN-2 may differ in their ability to form dimers. In the future, it will be important to determine if FLN-1 and FLN-2 can form hetero- or homodimers *in vivo*.

## Discussion

In this study, we use transcript sequencing and bioinformatics to identify and describe the gene structure of the *C. elegans* filamin orthologs, encoded by *fln-1* and *fln-2*. In addition, we use comparative homology modeling to predict the structures of the filamin repeats and the actin-binding domains. Taken together, our results suggest that the *C. elegans* filamin FLN-1 contains all features of the human filamins, including a well conserved actin-binding domain and 20 Ig-like filamin (IgFLN) domains separated into two rod regions. In contrast, FLN-2 is more divergent with an unusual ABD composed of three CH domains, and at least 23 Ig-like repeats interspersed with large regions of low homology. These results will help inform and validate future genetic and biochemical studies of filamin function, particularly those using *C. elegans* as a model system.

The gene structures of the two *C. elegans* filamins are quite divergent from each other. *fln-1* is smaller and is comprised of comparatively few large exons (Figure 1A), while *fln-2* has many small exons and is considerably longer (Figure 1B). Exons in *fln-1* appear to be modular units that contain one or more complete IgFLN repeats (Figure 3). Due to the smaller exon length, domains in *fln-2* are usually encoded by multiple exons (Figure 4). In both *fln-1* and *fln-2*, a few small exons appear to be alternatively spliced, but all are in frame and are not predicted to have much effect on the overall structure of the protein. It is likely that we have not detected all splice variants of *fln-1* and *fln-2*. *C. elegans* filamins appear to be extensively regulated through alternative splicing, and alternative transcriptional starts. Human filamins are also alternatively spliced, with consequences for ligand binding in the second rod domain [47]. These events are not known to produce large N- and C-terminal truncations like in *C. elegans* and *D. melanogaster*. Determining the function of these alternative filamin isoforms will be an interesting area for future research.

FLN-1 is structurally very similar to vertebrate filamins, particularly in the actin-binding domain and the second rod region. FLN-1 and Drosophila filamin *cheerio* both contain 20 IgFLN domains [33], [48]–[50]. The second rod region and the dimerization domain appear to be extremely well conserved between the worm, fly, and human filamins. In each case, the second rod domain consists of 8 IgFLN domains in the same order. The first rod domains of FLN-1 and *cheerio* lack four repeats, and have a lower overall conservation. It is possible that FLN-1 and *cheerio* represent ancestral filamins, which gave rise to vertebrate filamins through gene duplication events and modular expansion of the first rod domain [12]. We predict FLN-1 is likely to fold, interact and function in a manner similar to that of human filamins.

FLN-2 is a divergent form of filamin with low homology to vertebrate filamins. The closest homolog of FLN-2 outside of nematodes is the Drosophila filamin *jitterbug*. *jitterbug* shares many of the features of FLN-2, including an unusual actin-binding domain composed of three CH domains, and poorly conserved filamin repeats with long intervening sequences. *jitterbug* mutations cause seizures in affected flies, suggesting that *jitterbug* may have a role in the development or functioning of the nervous system [48], [49], [50]. Additionally, jitterbug is required for the mechanical response of notum tendon cells [51]. In the nematode, previously published transcript profiling data suggests full-length *fln-2a* is only expressed during embryogenesis, and the predominant larval form may be *fln-2e*, which lacks the ABD [34]. FLN-2 has not been investigated, but large-scale RNAi studies suggest it may play a role in molting [29], [30]. Consistent with this, we find FLN-2C localizes in punctate structures at the membrane of hypodermal cells, where it might play a structural or attachment role.

### FLN-1 and FLN-2 colocalize with F-actin *in vivo*

Filamins are an important class of actin crosslinking proteins that contribute to the stability of the actin cytoskeleton. We identified the actin binding domains of FLN-1 and FLN-2, and showed that they are highly conserved and structurally similar to mammalian filamins, particularly in areas critical for actin binding (Figure 5A–C). Importantly, we demonstrated that these putative ABDs co-localize with F-actin *in vivo*. This work is consistent with previous results which demonstrate that both FLN-1 and cheerio are required for integrity of the actin cytoskeleton in the reproductive system [28], [52], [53], [54].

Human disorders such as otopalatodigital syndrome (OPD) [55], periventricular heterotopia (PVH) [56], and boomerang dysplasia (BD) [57] can be caused by mutations in the filamin ABD, which modulates the affinity of filamin for F-actin. Therefore, a greater understanding of how the actin binding activity of filamin is regulated could be clinically relevant. Although biochemical experiments have shown *in vitro* binding of the filamin ABD by calmodulin [10] and phosphatidylinositol 4,5-bisphosphate (PIP_2_) [58], further studies are needed to determine how actin binding is regulated physiologically. Because the structural features of the ABD are preserved in *C. elegans*, the nematode may be a tractable model system to study this feature of filamin function.

### Homology modeling suggests FLN-1 may form dimers

Dimerization has long been considered critical for the actin-crosslinking function of filamin. Biochemical studies have identified the most C-terminal IgFLN domain as the primary dimerization domain [6], [9], [20], [45], [59], [60], [61]. Our homology modeling suggests that FLN-1 may form dimers in an orientation akin to vertebrate filamins, rather than in the manner of ddFLN, which lacks the hydrophobic dimerization interface [45]. FLN-1 dimerization could be important for its role in actin crosslinking in the nematode somatic gonad, especially in the uterus and spermatheca, where fluorescence microcopy reveals what appear to be V-shaped filamin clusters co-localized with branching actin filaments [28].

### Force sensation in the second rod domain of FLN-1

Filamin molecules can function as mechanical force sensors and have been shown to engage in distinct interactions depending on conformational state. The arrangement of filamin repeats has functional relevance and provides insight into how filamin may respond to mechanical stimuli [4], [33]. For example, studies using atomic force microscopy have demonstrated that the IgFLN repeats can withstand different amounts of force before unfolding [62], [63], and that this unfolding can interrupt or reveal binding sites. In particular, in the FLNA group IgFLN19–21, the N-terminal β-strand A of IgFLN20 is extended away from the rest of the sandwich, and participates structurally as a part of IgFLN21, which is nestled between IgFLN19 and IgFLN21 [4]. In this orientation, the N-terminus of IgFLN20 occupies the site where integrin preferentially binds. When this auto-inhibition is disrupted, the binding site is revealed, integrin binding affinity increases, and the length of the filamin molecule increases [4]. Structural data suggest that other IgFLN repeats in Rod 2 also interact with one another and may have similar effects on ligand binding and the cellular response to mechanical stress [33]. Our analysis suggests that the second rod domain of FLN-1 is well conserved, and that the domains are physically arranged as in the human filamins. The significant sequence and structural conservation suggests that FLN-1 may also exhibit differential interactions and auto-inhibition in response to changing mechanical conditions. Ongoing studies are testing the hypothesis that FLN-1 functions as a stretch-sensitive signaling scaffold *in vivo* [28].

## Materials and Methods

### Gene structure and sequencing of *fln-1* and *fln-2*

Wildtype N2 nematodes were obtained from the *Caenorhabditis* Genetics Center (Minneapolis, MN). Total RNA was extracted from mixed-stage N2 animal populations as described previously [25]. cDNA was prepared using SuperScript Reverse Transcriptase (Invitrogen; Carlsbad, CA, USA) and random hexamers as primers. cDNA sequencing was accomplished by amplifying regions of *fln-1* and *fln-2* by PCR, and subsequently cloning the amplicons into pGEM T-vector (Promega; Madison, WI, USA). Vector-specific primers (SP6 and T7) were used to sequence the inserts. Vector and poor quality sequences were trimmed, and the sequences were aligned to *C. elegans* genomic sequences using CLC bio Main Workbench version 5.2 (CLC bio; Cambridge, MA, USA). Tables S1A and S1B provide complete lists of primers used for sequencing *fln-1* and *fln-2* respectively. The amended *C. elegans* filamin gene names and structures have been submitted to WormBase (http://www.wormbase.org/) and are available as of the WS210 release [64]. Previous gene structure data is available in the referential WormBase WS205 release (http://ws205.wormbase.org/) and earlier releases.

### *fln-1* and *fln-2* expression

Sequences 5′ of each filamin transcript were amplified by PCR using N2 genomic DNA as a template. Each fragment was flanked by engineered *Hind*III and *Bam*HI restriction sites to facilitate cloning into the GFP expression vector pPD95.77 (Fire Vector Kit). Plasmids were isolated from *E. coli* and used for microinjection at an approximate concentration of 100 µg/mL. Transgenic strains were created by standard germline transformation [69] of wild type animals to create strains UN0824 *xbEx0824[fln-1c::gfp; ttx-3::rfp]* and UN0816 *xbEx0816[fln-2a,b::gfp]*. Strain UN1066 *xbEx0816[fln-2c::gfp]* was created by co-injection of *fln-2c* amplified from N2 genomic DNA using the high-fidelity Phusion polymerase (NEB, Ipswich, MA) and GFP and *unc-54* 3′ UTR sequences from pPD95.77. Strain UN0811 *xbEx0811[fln-1::gfp]* is previously described [28]. All primer sequences and cloning details available upon request. For visualization, transgenic animals were mounted on 1.5% agarose pads and imaged using differential interference contrast and epifluorescence microscopy with a Nikon Eclipse 80i microscope equipped with a SPOT RT3 CCD camera (Diagnostic Instruments; Sterling Heights, MI, USA).

### Phylogenetic trees

Filamin sequences from *C. elegans* [ACY40014.1 (ceFLN-1); ACY39993.1 (ceFLN-2)], *Drosophila melanogaster* [NP_524383.3 (dmCHER); NP_726234.3 (dmJBUG)], human [NP_001447.2 (hsFLNA); NP_001449.3 (hsFLNC); NP_001157789.1 (hsFLNB)], and Dictyostelium discoideum [XP_646669.1 (ddFLN)] were aligned using the ClustalW program [41]. A phylogenetic tree was constructed using the Neighbor Joining algorithm [35] and the BLOSUM62 matrix [65]. The phylogenetic tree of FLNA and FLN-1 repeats was constructed using CLC bio Main Workbench version 5.2.

### Domain identification

The assembled full-length sequences encoding FLN-1 and FLN-2 were used to identify the number and location of structural domains within the protein. IprScan, a PERL-based InterProScan utility [66], integrated into the SWISS-MODEL Workspace [36] was used to predict domains and functional features in the sequence. We also used SMART (Simple Modular Architecture Research Tool) [37], [38] to search for evolutionarily conserved protein domains, and scanned the sequences for SPF motifs that are often found near the end of IgFLN repeats [39].

### Homology modeling

For homology modeling of FLN-1 and FLN-2, the predicted amino acid sequences were divided into individual domains for alignment and template identification. We started with the Template Identification Tool incorporated into the SWISS-MODEL Workspace (http://swissmodel.expasy.org/), which uses BLAST to identify homologs in the SWISS-MODEL Template Library (SMTL) [40], and selects the best template structure based on sequence similarity, experimental quality, and secondary structure predictions. Domain sequences with less than 50% sequence identity to their selected template were realigned outside of the SWISS-MODEL Workspace using ClustalW [41] and visually inspected to ensure proper alignment prior to modeling. Modeling was accomplished using the SWISS-MODEL Workspace Alignment Mode, which is recommended for target and template sequences with 50–30% sequence identity [67]. Target-template pairs with less than 29% sequence identity were discarded. The resulting models and their templates were assessed globally for quality using the QMEAN server (http://swissmodel.expasy.org/qmean/) [42], and homology models scoring less than 0.500 were discarded. Molecular graphics images were produced using the UCSF Chimera package from the Resource for Biocomputing, Visualization, and Informatics at the University of California, San Francisco (supported by NIH P41 RR001081) [68].

### *In vivo* actin colocalization assay

To generate the *myo-3_P_*::VAB-10_ABD_::GFP control construct, the 2 kb *myo-3* promoter was amplified from N2 genomic DNA and cloned upstream of the cDNA encoding the first 1215 bp (405 aa) of the known actin-binding protein VAB-10, fusing it to GFP in the pPD95.77 vector (provided by A. Fire). Similarly, *myo-3_p_*::FLN-1_ABD_::GFP, the *myo-3_P_*::FLN-2_ABD_::GFP and the *myo-3_P_*::FLN-2_ABD(CH1+2)_::GFP constructs were produced by inserting cDNA representing either the first 792 bp of *fln-1*, 1076 bp of *fln-2* for the FLN-2_ABD_ construct or 704 bp of *fln-2* for the FLN-2_ABD(CH1+2)_ construct into the *Bam*HI and *Kpn*I sites of pPD95.77 vector containing the *myo-3* promoter. Transgenic lines carrying extrachromosomal arrays were generated via microinjection into N2 hermaphrodites as described [69] and given the following identifiers: UN0935 *xbEx0935[myo-3_P_::VAB-10_ABD_::GFP]*, UN0939 *xbEx0939[myo-3_P_::FLN-1_ABD_::gfp]*, UN0954 *xbEx0954[myo-3_P_::FLN-2_ABD_::gfp]* and UN1128 *xbEx1128[myo-3_P_::FLN-2_ABD_*_(CH1+2)_ *::gfp]*. *C. elegans* strains were grown on Nematode Growth Medium (NGM) plates fed with *E. coli* strain OP50 at 20°C unless otherwise stated [70].

To determine if the predicted ABD of FLN-1 and FLN-2 co-localize with actin *in vivo*, transgenic worms expressing fluorescent fusion proteins were fixed with 4% formaldehyde in PBS, placed on poly-L-lysine coated slides, and fixed in cold methanol and acetone. Fixed worms were stained with 0.4 U/mL of Texas Red-X phalloidin (Invitrogen) overnight at 4°C. The slides were washed, mounted, and viewed on a Nikon Eclipse 80i epifluorescence microscope. Images were documented with a SPOT RT3 CCD camera and SPOT Advanced software (Diagnostic Instruments; Sterling Heights, MI, USA.

## Supporting Information

Figure S1 **Sequencing of FLN-1 and FLN-2 transcripts.** Schematic representation of the gene structure of FLN-1 and FLN-2 with aligned sequencing results.
(TIF)

Figure S2 **FLN-1 IgFLN domain alignments.** Sequence alignment of all IgFLN repeats of *C. elegans* FLN-1 indicates strongly conserved regions similar to human filamins. Sequences are sorted by similarity, and group A repeats are indicated. The majority of the repeats ends with the SPF motif (black arrowheads), and contains conserved G, K, F, P, V, and Y residues. The majority consensus and the conservation level are indicated below the sequence alignment.
(TIF)

Figure S3 **Phylogenetic tree of FLNA and FLN-1 repeats.** Repeat classes are indicated with black bars and the corresponding letters. *C. elegans* and human repeats are labeled Ce and Hs, respectively. FLN-1 repeats are in orange, while FLNA repeats are in black. FLNA IgFLN24 has been classified as a group B repeat, and clusters with FLN-1 IgFLN20. FLNA group C repeats do not cluster with any FLN-1 repeats.
(TIF)

Table S1 **Sequencing primers.** Primers used for amplification of FLN-1 and FLN-2 transcripts for sequence. Vector-specific primers T7 and SP6 were also used for sequencing.
(XLS)

## References

[1] Stossel TP, Condeelis J, Cooley L, Hartwig JH, Noegel A (2001). Filamins as integrators of cell mechanics and signalling.. Nat Rev Mol Cell Biol.

[2] Feng Y, Walsh CA (2004). The many faces of filamin: a versatile molecular scaffold for cell motility and signalling.. Nat Cell Biol.

[3] Nakamura F, Heikkinen O, Pentikainen OT, Osborn TM, Kasza KE (2009). Molecular basis of filamin A-FilGAP interaction and its impairment in congenital disorders associated with filamin A mutations.. PLoS One.

[4] Lad Y, Kiema T, Jiang P, Pentikainen OT, Coles CH (2007). Structure of three tandem filamin domains reveals auto-inhibition of ligand binding.. EMBO J.

[5] Kiema T, Lad Y, Jiang P, Oxley CL, Baldassarre M (2006). The molecular basis of filamin binding to integrins and competition with talin.. Mol Cell.

[6] Nakamura F, Osborn TM, Hartemink CA, Hartwig JH, Stossel TP (2007). Structural basis of filamin A functions.. J Cell Biol.

[7] Gimona M, Djinovic-Carugo K, Kranewitter WJ, Winder SJ (2002). Functional plasticity of CH domains.. FEBS Lett.

[8] Lebart MC, Méjean C, Casanova D, Audemard E, Derancourt J (1994). Characterization of the actin binding site on smooth muscle filamin..

[9] Gorlin JB, Yamin R, Egan S, Stewart M, Stossel TP (1990). Human endothelial actin-binding protein (ABP-280, nonmuscle filamin): a molecular leaf spring.. J Cell Biol.

[10] Nakamura F, Hartwig JH, Stossel TP, Szymanski PT (2005). Ca2+ and calmodulin regulate the binding of filamin A to actin filaments.. J Biol Chem.

[11] Winder SJ, Hemmings L, Maciver SK, Bolton SJ, Tinsley JM (1995). Utrophin actin binding domain: analysis of actin binding and cellular targeting.. J Cell Sci 108 (Pt.

[12] Kesner BA, Milgram SL, Temple BR, Dokholyan NV (2010). Isoform divergence of the filamin family of proteins.. Mol Biol Evol.

[13] Robertson SP, Twigg SR, Sutherland-Smith AJ, Biancalana V, Gorlin RJ (2003). Localized mutations in the gene encoding the cytoskeletal protein filamin A cause diverse malformations in humans.. Nat Genet.

[14] Ithychanda SS, Hsu D, Li H, Yan L, Liu D (2009). Identification and characterization of multiple similar ligand-binding repeats in filamin: IMPLICATION ON FILAMIN-MEDIATED RECEPTOR CLUSTERING AND CROSS-TALK.. J Biol Chem.

[15] Loo DT, Kanner SB, Aruffo A (1998). Filamin binds to the cytoplasmic domain of the beta1-integrin. Identification of amino acids responsible for this interaction.. J Biol Chem.

[16] Onoprishvili I, Andria ML, Kramer HK, Ancevska-Taneva N, Hiller JM (2003). Interaction between the mu opioid receptor and filamin A is involved in receptor regulation and trafficking.. Mol Pharmacol.

[17] Ohta Y, Suzuki N, Nakamura S, Hartwig JH, Stossel TP (1999). The small GTPase RalA targets filamin to induce filopodia.. Proc Natl Acad Sci U S A.

[18] Marti A, Luo Z, Cunningham C, Ohta Y, Hartwig J (1997). Actin-binding protein-280 binds the stress-activated protein kinase (SAPK) activator SEK-1 and is required for tumor necrosis factor-alpha activation of SAPK in melanoma cells.. J Biol Chem.

[19] Davies PJ, Wallach D, Willingham M, Pastan I, Lewis MS (1980). Self-association of chicken gizzard filamin and heavy merofilamin.. Biochemistry.

[20] Weihing RR (1988). Actin-binding and dimerization domains of HeLa cell filamin.. Biochemistry.

[21] Jeon YJ, Choi JS, Lee JY, Yu KR, Ka SH (2008). Filamin B serves as a molecular scaffold for type I interferon-induced c-Jun NH2-terminal kinase signaling pathway.. Mol Biol Cell.

[22] Tigges U, Koch B, Wissing J, Jockusch BM, Ziegler WH (2003). The F-actin cross-linking and focal adhesion protein filamin A is a ligand and in vivo substrate for protein kinase C alpha.. J Biol Chem.

[23] Corsi AK (2006). A biochemist's guide to Caenorhabditis elegans.. Anal Biochem.

[24] Gettner SN, Kenyon C, Reichardt LF (1995). Characterization of beta pat-3 heterodimers, a family of essential integrin receptors in C. elegans.. J Cell Biol.

[25] Cram EJ, Clark SG, Schwarzbauer JE (2003). Talin loss-of-function uncovers roles in cell contractility and migration in C. elegans.. J Cell Sci.

[26] Cox EA, Hardin J (2004). Sticky worms: adhesion complexes in C. elegans.. J Cell Sci.

[27] Lundquist EA (2006). Small GTPases.. WormBook.

[28] Kovacevic I, Cram EJ (2010). FLN-1/filamin is required for maintenance of actin and exit of fertilized oocytes from the spermatheca in C. elegans.. Dev Biol.

[29] Kamath RS, Fraser AG, Dong Y, Poulin G, Durbin R (2003). Systematic functional analysis of the Caenorhabditis elegans genome using RNAi.. Nature.

[30] Frand AR, Russel S, Ruvkun G (2005). Functional genomic analysis of C. elegans molting.. PLoS Biol.

[31] Conrad R, Thomas J, Spieth J, Blumenthal T (1991). Insertion of part of an intron into the 5′ untranslated region of a Caenorhabditis elegans gene converts it into a trans-spliced gene.. Mol Cell Biol.

[32] Blumenthal T (2005). Trans-splicing and operons..

[33] Heikkinen OK, Ruskamo S, Konarev PV, Svergun DI, Iivanainen T (2009). Atomic structures of two novel immunoglobulin-like domain pairs in the actin cross-linking protein filamin.. J Biol Chem.

[34] Gerstein MB, Lu ZJ, Van Nostrand EL, Cheng C, Arshinoff BI (2010). Integrative analysis of the Caenorhabditis elegans genome by the modENCODE project.. Science.

[35] Saitou N, Nei M (1987). The neighbor-joining method: a new method for reconstructing phylogenetic trees.. Mol Biol Evol.

[36] Arnold K, Bordoli L, Kopp J, Schwede T (2006). The SWISS-MODEL workspace: a web-based environment for protein structure homology modelling.. Bioinformatics.

[37] Letunic I, Doerks T, Bork P (2009). SMART 6: recent updates and new developments.. Nucleic Acids Res.

[38] Schultz J, Milpetz F, Bork P, Ponting CP (1998). SMART, a simple modular architecture research tool: identification of signaling domains.. Proc Natl Acad Sci U S A.

[39] Sjekloca L, Pudas R, Sjoblom B, Konarev P, Carugo O (2007). Crystal structure of human filamin C domain 23 and small angle scattering model for filamin C 23-24 dimer.. J Mol Biol.

[40] Kiefer F, Arnold K, Kunzli M, Bordoli L, Schwede T (2009). The SWISS-MODEL Repository and associated resources.. Nucleic Acids Res.

[41] Chenna R, Sugawara H, Koike T, Lopez R, Gibson TJ (2003). Multiple sequence alignment with the Clustal series of programs.. Nucleic Acids Res.

[42] Benkert P, Tosatto SC, Schomburg D (2008). QMEAN: A comprehensive scoring function for model quality assessment.. Proteins.

[43] Sawyer GM, Clark AR, Robertson SP, Sutherland-Smith AJ (2009). Disease-associated substitutions in the filamin B actin binding domain confer enhanced actin binding affinity in the absence of major structural disturbance: Insights from the crystal structures of filamin B actin binding domains.. J Mol Biol.

[44] Bosher JM, Hahn BS, Legouis R, Sookhareea S, Weimer RM (2003). The Caenorhabditis elegans vab-10 spectraplakin isoforms protect the epidermis against internal and external forces.. J Cell Biol.

[45] Pudas R, Kiema TR, Butler PJ, Stewart M, Ylanne J (2005). Structural basis for vertebrate filamin dimerization.. Structure.

[46] Nakamura F, Pudas R, Heikkinen O, Permi P, Kilpelainen I (2006). The structure of the GPIb-filamin A complex.. Blood.

[47] Pentikainen U, Jiang P, Takala H, Ruskamo S, Campbell ID (2011). Assembly of a filamin four domain fragment and the influence of splicing variant-1 on the structure.. J Biol Chem.

[48] Hekmat-Scafe DS, Dang KN, Tanouye MA (2005). Seizure suppression by gain-of-function escargot mutations.. Genetics.

[49] Hekmat-Scafe DS, Lundy MY, Ranga R, Tanouye MA (2006). Mutations in the K+/Cl- cotransporter gene kazachoc (kcc) increase seizure susceptibility in Drosophila.. J Neurosci.

[50] Song J, Tanouye MA (2006). Seizure suppression by shakB2, a gap junction mutation in Drosophila.. J Neurophysiol.

[51] Olguin P, Glavic A, Mlodzik M (2011). Intertissue mechanical stress affects frizzled-mediated planar cell polarity in the Drosophila notum epidermis.. Curr Biol.

[52] Sokol NS, Cooley L (1999). Drosophila filamin encoded by the cheerio locus is a component of ovarian ring canals.. Curr Biol.

[53] Li MG, Serr M, Edwards K, Ludmann S, Yamamoto D (1999). Filamin is required for ring canal assembly and actin organization during Drosophila oogenesis.. J Cell Biol.

[54] Sokol NS, Cooley L (2003). Drosophila filamin is required for follicle cell motility during oogenesis.. Dev Biol.

[55] Hidalgo-Bravo A, Pompa-Mera EN, Kofman-Alfaro S, Gonzalez-Bonilla CR, Zenteno JC (2005). A novel filamin A D203Y mutation in a female patient with otopalatodigital type 1 syndrome and extremely skewed X chromosome inactivation.. Am J Med Genet A.

[56] Fox JW, Lamperti ED, Eksioglu YZ, Hong SE, Feng Y (1998). Mutations in filamin 1 prevent migration of cerebral cortical neurons in human periventricular heterotopia.. Neuron.

[57] Bicknell LS, Morgan T, Bonafe L, Wessels MW, Bialer MG (2005). Mutations in FLNB cause boomerang dysplasia.. J Med Genet.

[58] Furuhashi K, Inagaki M, Hatano S, Fukami K, Takenawa T (1992). Inositol phospholipid-induced suppression of F-actin-gelating activity of smooth muscle filamin.. Biochem Biophys Res Commun.

[59] McCoy AJ, Fucini P, Noegel AA, Stewart M (1999). Structural basis for dimerization of the Dictyostelium gelation factor (ABP120) rod.. Nat Struct Biol.

[60] Popowicz GM, Muller R, Noegel AA, Schleicher M, Huber R (2004). Molecular structure of the rod domain of dictyostelium filamin.. J Mol Biol.

[61] Stossel TP, Condeelis J, Cooley L, Hartwig JH, Noegel A (2001). Filamins as integrators of cell mechanics and signalling.. Nat Rev Mol Cell Biol.

[62] Furuike S, Ito T, Yamazaki M (2001). Mechanical unfolding of single filamin A (ABP-280) molecules detected by atomic force microscopy.. FEBS Lett.

[63] Yamazaki M, Furuike S, Ito T (2002). Mechanical response of single filamin A (ABP-280) molecules and its role in the actin cytoskeleton.. J Muscle Res Cell Motil.

[64] Harris TW, Antoshechkin I, Bieri T, Blasiar D, Chan J (2010). WormBase: a comprehensive resource for nematode research.. Nucleic Acids Res.

[65] Henikoff S, Henikoff JG (1992). Amino acid substitution matrices from protein blocks.. Proc Natl Acad Sci U S A.

[66] Zdobnov EM, Apweiler R (2001). InterProScan–an integration platform for the signature-recognition methods in InterPro.. Bioinformatics.

[67] Bordoli L, Kiefer F, Arnold K, Benkert P, Battey J (2009). Protein structure homology modeling using SWISS-MODEL workspace.. Nat Protoc.

[68] Pettersen EF, Goddard TD, Huang CC, Couch GS, Greenblatt DM (2004). UCSF Chimera--a visualization system for exploratory research and analysis.. J Comput Chem.

[69] Mello CC, Kramer JM, Stinchcomb D, Ambros V (1991). Efficient gene transfer in C.elegans: extrachromosomal maintenance and integration of transforming sequences.. EMBO J.

[70] Brenner S (1974). The genetics of Caenorhabditis elegans.. Genetics.

