# Supplementary figures and images for: Structural and Functional Evaluation of C. elegans Filamins FLN-1 and FLN-2
Source: PLoS One. 2011 Jul 25;6(7):e22428. doi: 10.1371/journal.pone.0022428 (PMC3143143; doi:10.1371/journal.pone.0022428)

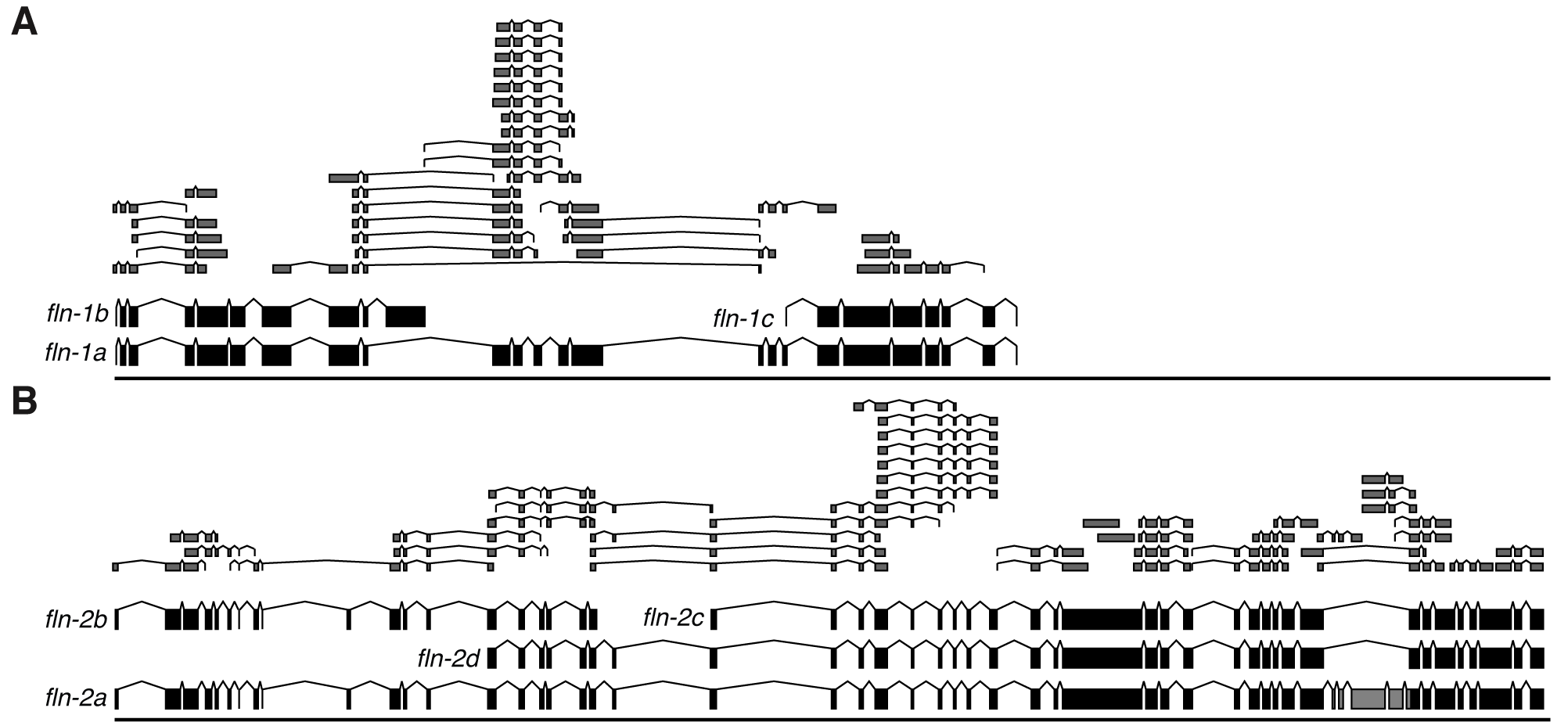

Supplement: Figure S1 — Sequencing of FLN-1 and FLN-2 transcripts. Schematic representation of the gene structure of FLN-1 and FLN-2 with aligned sequencing results. (TIF) [file pone.0022428.s001.tif]

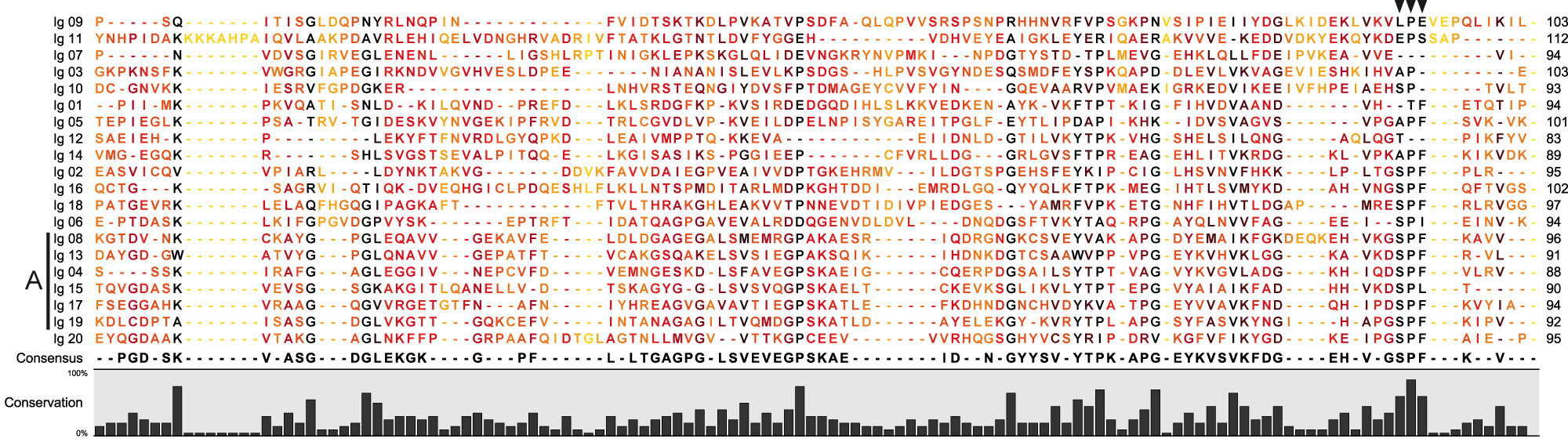

Supplement: Figure S2 — FLN-1 IgFLN domain alignments. Sequence alignment of all IgFLN repeats of C. elegans FLN-1 indicates strongly conserved regions similar to human filamins. Sequences are sorted by similarity, and group A repeats are indicated. The majority of the repeats ends with the SPF motif (black arrowheads), and contains conserved G, K, F, P, V, and Y residues. The majority consensus and the conservation level are indicated below the sequence alignment. (TIF) [file pone.0022428.s002.tif]

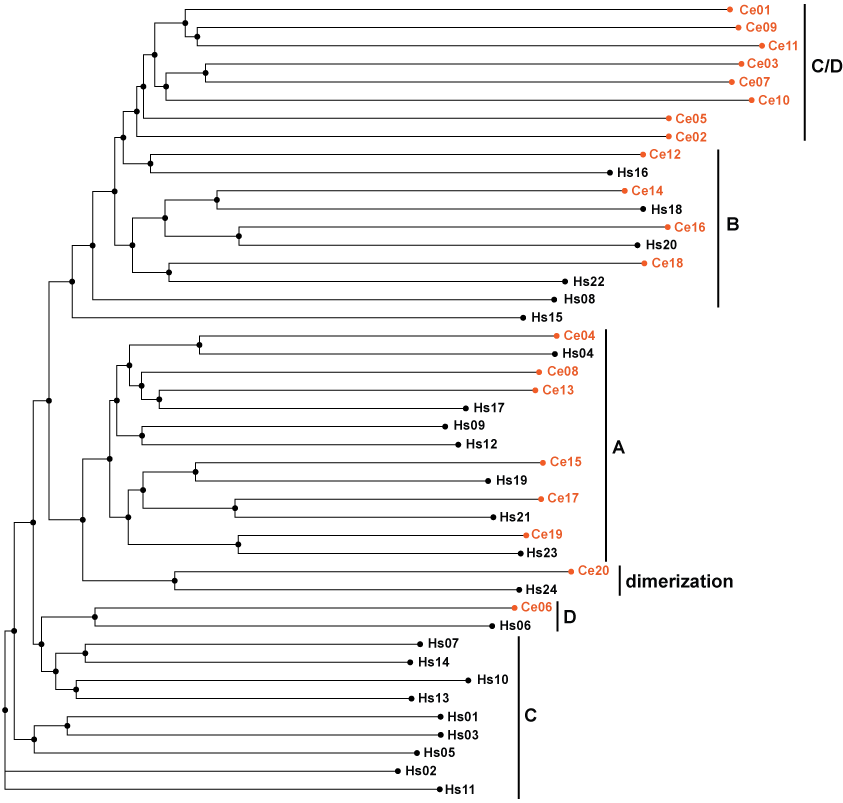

Supplement: Figure S3 — Phylogenetic tree of FLNA and FLN-1 repeats. Repeat classes are indicated with black bars and the corresponding letters. C. elegans and human repeats are labeled Ce and Hs, respectively. FLN-1 repeats are in orange, while FLNA repeats are in black. FLNA IgFLN24 has been classified as a group B repeat, and clusters with FLN-1 IgFLN20. FLNA group C repeats do not cluster with any FLN-1 repeats. (TIF) [file pone.0022428.s003.tif]
